# Dogs Detecting COVID-19 From Sweat and Saliva of Positive People: A Field Experience in Mexico

**DOI:** 10.3389/fmed.2022.837053

**Published:** 2022-04-01

**Authors:** Juan Manuel Mancilla-Tapia, Victoria Lozano-Esparza, Adrián Orduña, Reyna Fabiola Osuna-Chávez, Ramón Enrique Robles-Zepeda, Blayra Maldonado-Cabrera, Jorge Rubén Bejar-Cornejo, Iván Ruiz-León, Carlos Gabriel González-Becuar, Anna Hielm-Björkman, Ana Novelo-González, Victor Manuel Vidal-Martínez

**Affiliations:** ^1^Canine Training Center Obi-K19, Hermosillo, Mexico; ^2^División de Ciencias Biológicas y de la Salud, Departamento de Agricultura y Ganadería, Universidad de Sonora, Hermosillo, Mexico; ^3^Hospital General del Estado de Sonora, Secretaria de Salud Pública del Estado de Sonora, Hermosillo, Mexico; ^4^Department of Clinical Veterinary Sciences, Faculty of Veterinary Medicine, University of Helsinki, Helsinki, Finland; ^5^Laboratorio de Patología Acuática, Departamento de Recursos del Mar, Centro de Investigación y de Estudios Avanzados del Instituto Politécnico Nacional Unidad Mérida, Mérida, Mexico

**Keywords:** SARS-CoV-2, COVID-19, sniffing dogs, pathogen detection, dog training, olfactory detection, bio-detection, Mexico

## Abstract

**Context:**

Molecular tests are useful in detecting COVID-19, but they are expensive in developing countries. COVID-19-sniffing dogs are an alternative due to their reported sensitivity (>80%) and specificity (>90%). However, most of the published evidence is experimental, and there is a need to determine the performance of the dogs in field conditions. Hence, we aimed to test the sensitivity and specificity of COVID-19-sniffing dogs in the field.

**Methods:**

We trained four dogs with sweat and three dogs with saliva of COVID-19-positive patients, respectively, for 4.5 months. The samples were obtained from a health center in Hermosillo, Sonora, with the restriction to spend 5 min per patient. We calculated sensitivity, specificity, and their 95% confidence intervals (CI).

**Results:**

Two sweat-sniffing dogs reached 76 and 80% sensitivity, with the 95% CI not overlapping the random value of 50%, and 75 and 88% specificity, with the 95% CI not overlapping the 50% value. The 95% CI of the sensitivity and specificity of the other two sweat dogs overlapped the 50% value. Two saliva-sniffing dogs had 70 and 78% sensitivity, and the 95% CI of their sensitivity and specificity did not overlap the 50% value. The 95% CI of the third dog's sensitivity and specificity overlapped the 50% value.

**Conclusion:**

Four of the six dogs were able to detect positive samples of patients with COVID-19, with sensitivity and specificity values significantly different from random in the field. We considered the performance of the dogs promising because it is reasonable to expect that with gauze exposed for a longer time to sweat and saliva of people with COVID-19, their detection capacity would improve. The target is to reach the sensitivity range requested by the World Health Organization for the performance of an antigen test (≥80% sensitivity, ≥97% specificity). If so, dogs could become important allies for the control of the COVID-19 pandemic, especially in developing countries.

## Introduction

Since the massive spread of severe acute respiratory syndrome coronavirus 2 (SARS-CoV-2) began in late 2019, it has been extremely important to develop fast and reliable detection methods to control the pandemic (1). In many countries, and especially in those with limited economic resources, it has been very difficult to implement mass virus detection programs (2–4). This is because the recommended approaches are high-cost molecular methods such as quantitative polymerase chain reaction (qPCR). Less expensive antigen tests are still non-accessible to developing countries due to their cost as well as difficulty in implementation due to the lack of proper facilities and biosafety systems in place (2, 3). This is a reason why many countries are interested in the use of dogs to detect people infected by SARS-CoV-2. Dogs have more than 220 million olfactory receptors, while humans only have five million (i.e., 2.27% of the number present in dogs) (5). This olfactory potential gives dogs capabilities that can be used to search for people; to detect narcotics and explosives; and to detect diseases such as cancer, malaria, and epilepsy (6–11). Based on these capabilities, it has been inferred that these dogs could help to detect infectious diseases because previous studies have concluded that volatile organic compounds (VOC) produced by infected organisms can be detected by canine olfactory organs (7, 12, 13). In this context, dogs trained to detect SARS-CoV-2 appear as a very promising and cost-effective alternative, as there is evidence of their ability to detect and differentiate VOCs produced by people with COVID-19 (3, 14, 15).

Inspired by the previous work mentioned above, we developed a methodology to train dogs to detect people with COVID-19. In November 2020, we were still training the dogs when the second wave of SARS-CoV-2 infection arrived at our city (Hermosillo, Sonora). At that time, the local government asked us to take the dogs into the field for detection. For this reason, we were allowed to obtain the samples of infected and non-infected people at the Anticipa Health Center in Hermosillo, which makes the present study one of the few attempts to test the performance of COVID-19-sniffing dogs in real life (as opposed to in an experimental setting). However, to get the samples we had to follow the rules of the health providers, who requested that we interact with their patients for the shortest possible time. Thus, we were allowed to collect our three samples; body sweat, axillary sweat, and saliva samples, during only 5 min per patient, which then produced a low level of impregnation of the provided pieces of gauze. We thereby anticipated some decrease in the performance of the dogs but considered it important to share the present results to show the potential of COVID-19-sniffing dogs for middle-income countries like Mexico even under these challenging circumstances.

Our original hypothesis was that our training procedure enables the dogs to detect people with COVID-19. To test this hypothesis, our objective was to determine the sensitivity and specificity of COVID-19-sniffing dogs in field conditions and to compare their performance with the results obtained from the reverse transcription polymerase chain reaction (RT-PCR) and antigen tests for the same patients.

## Materials and Methods

### Sampling Sites

We used sweat and saliva samples of COVID-19-positive and COVID-19-negative humans for dog detection. A convenience sampling methodology was used, obtaining samples from a small number of places during the training and the field study. These institutions were chosen because of their willingness to provide samples. All the samples were obtained from Hermosillo, Sonora, or nearby localities. Specifically, the samples were obtained from the Anticipa Health Center (Hermosillo) (1,932 samples), the General Hospital of the State of Sonora (Hermosillo) (eight samples), Centro Anticipa (Empalme) (eight samples), the Myco textile factory (Empalme) (48 samples), and the University of Sonora (Hermosillo) (eight samples). These samples were used to train the dogs. All the samples were obtained in Sonora between September 8, 2020, and March 31, 2021.

### Procedures for Obtaining Sweat and Saliva Samples From COVID-19-Positive and COVID-19-Negative Patients

Before sampling, each patient was asked for her/his willingness to participate in the research, and in the case of a positive answer, a briefing on the objectives of the project and the subsequent use of the samples and epidemiological information was provided. The patient was then provided an informed consent form to read and sign, accepting her/his participation in the project (Supplementary Material 1). At the same time, a questionnaire was used to obtain epidemiological data on symptoms and medical history of the patient. The questionnaire collected the following information: full name; age; sex; diagnosed chronic diseases; alcohol, cigarette, or drug use; headache; diarrhea; fever; loss of taste; loss of smell; cough; runny nose; sore throat; body ache; chest pain; nausea; days with symptoms; treatment and days on medication (if provided); and contact with confirmed COVID-19-positive people.

The positive or negative status of each patient was confirmed by RT-PCR performed by the State Laboratory of Public Health and/or by the Panbio COVID-19 Ag Rapid Test Device (Abbott^®^) antigen test, and in some cases by both tests (see Supplementary Material 2). All positive samples came from symptomatic patients or individuals with mild symptoms such as a cold, fever, headache, and/or diarrhea. These data were registered in a database that can be requested from Juan Mancilla-Tapia. The selected samples were from patients who had had symptoms for ≤ 9 days, with a preference for early stage infected who had had mild symptoms for 1–3 days. We also recorded the medications used by each patient (paracetamol, ibuprofen, or other cold medications). Thus, the inclusion criteria for COVID-19-positive patients were (1) age range between 18 and 60 years old, (2) ≤ 9 days of symptoms, and (3) positive for SARS-CoV-2 confirmed by RT-PCR or by the Panbio COVID-19 Ag Rapid Test Device antigen test.

Most of the negative samples were from mildly symptomatic patients, with symptoms like diarrhea, headache, fever, and/or a cold. However, the inclusion criteria for the COVID-19-negative sample collection did not include the most specific symptoms of COVID-19 such as loss of smell or taste or respiratory problems. Negative samples were also obtained from asymptomatic patients. All negative samples had a negative RT-PCR or Panbio COVID-19 Ag Rapid Test Device antigen test result.

The samples for RT-PCR and the antigen test were obtained from the throat and nasopharynx of all patients following standard procedures recommended by the Mexican health authority (16). The RT-PCR tests were performed by the Laboratory of Molecular Biology of the University of Sonora following the instructions and using the kits recommended by the Mexican health authorities (17). Adequate personal protective equipment was used while collecting the throat and nasopharynx samples and performing the antigen tests. The antigen tests were performed following the instructions of the device manufacturer. All sweat samples, regardless of whether the tests indicated they were positive or negative for SARS-CoV-2, were handled by following the safety measures recommended by the Mexican health authorities (16). Thus, we used KN 95 face masks, nitrile gloves, and all the personal protective equipment recommended by Mexican health authorities. During sample collection, the biological risk was considered “greater than the minimum” because the patients took their own samples under our technical supervision. Technicians did not take samples from patients. This process was approved by the Bioethics and Safety Committee of the University of Sonora. The Mexican health authorities requested that the whole process of providing the briefing, obtaining the consent, applying the epidemiological questionnaire and obtaining the sweat and saliva samples should take no more than 5 min per patient. To collect the sweat samples, each patient was given a pair of non-sterile, dust-free nitrile gloves and a resealable Ziploc^®^ bag containing two 5 cm high, 8 cm diameter translucent glass flasks with metallic caps that had been sterilized in an autoclave and under UVC light, six pieces of new, sterile Jaloma^®^ odorless gauze (10 cm × 10 cm), and four sterile dental swabs. The patient was asked to rub her/his neck, face, and forearms for 1 min with two pieces of gauze on the left half of her/his head, and then wipe the other two pieces of gauze on the right half of her/his head. Subsequently, the patient was asked to place two dental swabs in her/his mouth and one dental swab under each armpit for 1 min. After this time, the patient was instructed to insert the pieces of gauze and swabs with sweat samples into the glass flask, to close it, and to place it back in the resealable bag. Briefly, to collect the saliva sample, each patient was given a pair of non-sterile, dust-free nitrile gloves and a resealable Ziploc^®^ bag containing two 5 cm high, 8 cm diameter translucent glass flasks with metallic caps sterilized in an autoclave and under UVC light, plus two sterile dental swabs. The patient was asked to place two dental swabs in her/his mouth for 1 min. Next, the patient was instructed to insert the dental swabs into the glass flask, to close it, and to place it back in the resealable bag. Again, this process was short, as it took <3 min per patient. No fixatives were added to the saliva samples for training of the dogs. The samples were then transported in coolers to the laboratory and kept there at 18°C until they were used. In the case of the sweat and saliva samples for the field study, the procedure was the same as described above, but the samples were transported immediately to the second floor of the Anticipa Health Center where the dogs and line-up were allocated for COVID-19 detection.

### Selection of Canines for Training and Target Odor

Nine dogs were originally selected for training. Dogs without previous training are known as “green dogs” because they have the instinct and performance to pass the different training phases, but they have never been exposed to odors that would affect detection of COVID-19. Six of the nine dogs successfully completed their training. They were a 1-year-old female puppy who was originally in the training process to be an epilepsy detection canine (Leia), two 2-year-old German Shepherd males (Mike and Sam) with no prior training (green dogs from narcotic line), a 1-year-old Belgian Malinois (Krilling) with no prior training (a green dog from narcotic and sport line), and two 2-year-old Belgian Malinois (Harry and Spaidy) without prior training (green dogs from narcotic and sport line). The rest of the dogs did not complete their training for various reasons, such as sickness (not COVID-19) or inability to perform as sniffing dogs. For these reasons, they were allocated to different places as companion pets and are not considered further in this study.

Jendrny et al. (3), Grandjean et al. (14), and Essler et al. (15) have successfully proved that dogs can discriminate between COVID-19-positive and COVID-19-negative individuals based on exposure to different body metabolites excreted through the breath, urine, tears, saliva, feces, and sweat. Grandjean et al. (14) specifically suggested that dogs can detect VOC produced by humans and excreted through their sweat. We concur that dogs have the capacity to detect VOC in human sweat. Our goal was to determine whether this capacity is high enough to be used as a preliminary but effective test to discriminate COVID-19-positive and COVID-19-negative individuals. Thus, we assumed that the odor the dogs are detecting are the metabolites produced by infected humans, and that the dogs are able to discriminate this odor from potentially confounding odors such as those produced by deodorants and anti-transpirants, among many others.

### Training Dogs for Odor Detection From Sweat and Saliva

The canine training to detect SARS-CoV-2 lasted ~12 weeks in the laboratory, with two sessions per day, 5 days a week (Monday to Friday) and one session on Saturday morning. These dogs were the first generation to be trained for COVID-19 detection and were trained exclusively on corporal (as opposed to axillary) sweat samples. All the experiments were performed in air-conditioned experimental facilities at the Obi-K19 canine training center in Hermosillo, Sonora, because the environmental temperature (25–36°C) and low humidity of Hermosillo (https://es.weatherspark.com/m/2272/10/Tiempo-promedio-en-octubre-en-Hermosillo-México) were expected to affect the dogs' performance. Similar negative influences in the performance of dogs have been reported elsewhere (18). Our objective was, that at the end of the training process, the dogs would be able to discriminate the odor (VOC) of the sweat or saliva of a person with COVID-19 from the sweat or saliva of an uninfected person. The dog training plan had three phases: target odor association, discrimination, and training evaluation.

The target odor association training lasted 3 weeks. Before exposing the dog to the odor produced by people with COVID-19, the dogs were first taught how to use their sense of smell in a targeted manner. Thus, the dogs were trained to mark a toy-type odor. This process consisted of the dog learning two main aspects: the association of marking the toy-type odor with something positive, using the toy as a reward, and training the dog to look for a piece of the toy placed in a stainless-steel box. The toy used for training the dogs is known as the Kong^®^, and each time they found it in the box, they were allowed to play with it for 3–5 min as a positive reinforcer of behavior. It is important to emphasize that stainless steel was used in all these materials because they do not keep odors. Once the dogs had learned to mark the box with the Kong, they were moved on to the next phase of training.

Once the dogs associated the act of finding something among several boxes with a reward, the objective of reinforcing the desired behavior was achieved. The duration of this phase was 2 weeks. In this phase, a sweat sample was used for the first time. The procedure consisted of allocating a gauze of a COVID-19-positive patient into a stainless-steel salt shaker alongside a piece of Kong because it was an odor the dogs already knew. The purpose of this exercise was for the dog to become familiar with the odor of a sweat sample from a COVID-19-positive patient and to associate it with the reward. This phase of training lasted 4 days. The last part of this phase was the removal of the piece of Kong to leave only the gauze and determine whether the canine marked it correctly. This second step lasted about 10 days. Once the gauze had been used, it was discarded as hazardous biological infectious waste (HBIW). It is worth mentioning that we discovered that the gauze we were using (Protect^®^, Mexico City, Mexico) had an odor added during the production process. For this reason, we decided to change the gauze, and thereafter we used only a completely odorless gauze by Jaloma^®^ (Guadalajara, Mexico).

In the discrimination phase, we worked with the dogs Harry, Sam, and Mike for 9 weeks and Leia for 11 weeks. The odor of the positive sample was increased by including two or three pieces of gauze for 2 weeks. After that, the discrimination of negative samples began. Two salt shakers were used, one containing sweat swabs from RT-PCR-positive patients and one containing sweat swabs from RT-PCR-negative patients. These samples were placed randomly by a trained assistants in a stainless-steel line with four holes, so neither the trainer nor the researcher knew where the positive samples were. In a subsequent exercise, two salt shakers with negative samples and one with a positive sample were placed, allocating the positive sample in the first hole for the first and second checkups. The positive sample was then moved to the second and third hole in subsequent check-up opportunities. This approach was followed to train the dogs to follow a search sequence. Finally, three salt shakers with negative samples and one with a positive sample were introduced. At this stage, the positive sample was introduced into the first hole during the first and second checkups, moving it to positions two, three, and four in subsequent checkups to reinforce the search sequence to the dog. Subsequently, the dogs were presented with three salt shakers with two negative pieces of gauze (from the same patient) each and a salt shaker with five positive pieces of gauze (from the same patient). The number of positive pieces of gauze was decreased until the dog marked and discriminated samples with the same number of positive and negative pieces of gauze in the salt shakers.

During the training evaluation, we tested the ability of the dogs to distinguish between COVID-19-positive and COVID-19-negative samples. This evaluation was double blind and lasted 2 weeks. Each salt shaker contained the same number of gauze pieces. We used an odor line-up with four holes, presenting one positive sample and three negative samples sequentially for each trial. The dogs were allowed to make a first check of all the samples, followed by a second check when the trainer asked the dog to look for the positive sample. The task of this phase was for the dog to mark five positive samples correctly and consecutively. Once the dog was able to do this, she/he was considered trained. After completing these phases, the dogs entered field work.

A second generation of dogs (Spaidy, Krilling, and Leia) was trained exclusively with saliva of SARS-CoV-2-positive samples from patients with loss of taste and smell. These samples came from the COVID-19 wards of the General Hospital of the State of Sonora and the Cima Hospital of the city of Hermosillo. The procedure to obtain the samples was similar to the one used for sweat samples, with some slight differences.

The second-generation dog training methodology changed slightly. First, instead of the Kong toy, we used food to induce the association of the dog with the saliva sample. Second, we did not include food in the salt shaker together with the COVID-19-positive saliva sample. During the scent association phase, the dogs were only fed on a table that had a flask containing the positive sample. This helped them associate the odor of the COVID-19-positive saliva sample with a reward, in this case food. A second empty vial was then added, and the dog only ate when she/he placed her/his nose in the salt shaker with the positive sample. However, after using food for 3 weeks we decided to go back to the Kong as a reward with two of the dogs (Spaidy and Krilling) because they were originally trained with the toy and performed better than those trained with food. The use of saliva samples reduced the training time to 10 weeks, and after this time the dogs were ready to enter field work. These dogs learned to detect the odor faster than the previous generation due to exposure to samples from positive patients with many of the typical symptoms of COVID-19.

### Trial Procedure in Field Conditions and Statistical Analysis

All the samples (positive and negative) for the trials were obtained at the Anticipa Health Center. This Health Center is a two-level building where the samples from patients were obtained on the ground level and immediately taken upstairs to be allocated in the line-ups. For each trial, we used two identical odor lines. Each stainless-steel odor line had four holes, but due to the shortage of samples, we used only one salt shaker containing a positive sample and one salt shaker with a negative sample. The other two holes in the line had empty salt shakers and were not considered in the statistical calculations. The positive or negative status of the patients to SARS-CoV-2 was based on the Ag test result in 52% of the cases. The positive or negative status of the remaining 48% of the samples was based on symptoms related to COVID-19 and corroborated by the RT-PCR 2 weeks later. Each dog was passed through all the samples in a line a first time for recognition, and after that she/he was passed a second time for definitive detection in the same line. The same procedure was repeated for the second line. In this way, the line-up used in the present study was like that the one described by Kaesler et al. (19). This method apparently improves discriminability by imposing additional memory demand on each dog: They must encode information and the line-up position for the second round. Thus, we slightly modified the methodology proposed by Grandjean et al. (14) as follows: For each trial in a line-up, the dog sniffed each of the two salt shakers for a first time. In the second round, the dog handler asked the dog to look for the positive sample, and the salt shaker the dog marked was considered the definitive identification decision for that trial. For each trial, in contrast to the previous training phases, a sample here comprised two sterile pieces of gauze exposed for 1 min to axillary sweat and one piece of gauze exposed for 1 min to corporal sweat of a patient. These gauzes were introduced in a plastic jar which was sealed for 1–5 min while the technician was setting up the samples. Maintaining the plastic jar sealed for a couple of minutes allowed the odor to impregnate better into the sample. These plastic jars with the sample were allocated in an autoclaved stainless-steel saltshaker 10 cm tall and 7 cm in diameter. The negative sample for that trial was obtained from people present in the health center where the positive samples were obtained and processed in the same way as the positive ones. For each trial, new fresh positive and negative samples were always used, and none of the previous samples were used again. The positive and negative samples were allocated randomly by the data recorder, and neither the dog handler nor the dog knew where the positive samples were. In fact, both the dog handler and the dog were looking in a different direction when the salt shakers were allocated in the line-up (double-blind strategy). The recorder indicated that the line-up was ready and it was at this moment that both the dog handler and the dog faced the line-up. Once the dog sniffed all the salt shakers and marked one (by sitting, or laying on it) the dog handler made a signal (upright closed fist) to indicate that the trial had finished. The data recorder indicated verbally whether the mark was correct, and if so, the dog handler immediately rewarded the dog with the Kong, allowing him/her 2–3 min to play with the toy. The exposure procedure of the dogs to saliva samples was the same as for sweat samples, with the only exception that each plastic jar with two dental swabs positive for SARS-CoV-2 was allocated and opened in a sterilized salt shaker. The negative samples were two clean dental swabs in a plastic jar that was introduced and opened in a sterilized salt shaker. The testing period lasted for 12 weeks, during which time none of the dogs showed disease signs.

Sensitivity and specificity were calculated as recommended by Johnen et al. (20) because the line-up was a mixture of simultaneous and sequential line-up as explained above. Each trial was a typical Bernoulli experiment where the probability of success or failure is 50% (like the flip of a coin). Thus, after many Bernoulli experiments exposing the dogs to COVID-19-positive and COVID-19-negative samples, we were able to calculate 95% confidence intervals (CI). If a 95% CI does not overlap 50%, which is the randomness region, that 95% CI could be considered significantly different from a random choice. Thus, we calculated 95% CI of the sensitivity and specificity with Clopper–Pearson's method, using the package epiR (https://cran.r-project.org/web/packages/epiR/index.html), and considered significant those 95% CI that did not overlap ≤ 50% sensitivity and specificity values. We followed the procedures recommended by Trevethan (21) to calculate sensitivity and specificity. The minimum number of samples to be sniffed for an adequate study power was calculated assuming a 15% of prevalence of SARS-CoV-2 in the population. This was a rather conservative estimation of prevalence since by November 2020, we were in the middle of the second wave of SARS-CoV-2 at Hermosillo. The probability of type 1 error, determining that there is a difference when such difference does not actually exist, was established at 0.05. The power of the analysis to detect a difference between groups when such a difference exist was assumed to be 80%. All the calculations for the number of samples to be sniffed were made with ClinCalc.com (https://clincalc.com/stats/samplesize.aspx). These calculations gave us a sample size of 94 to have an adequate study power.

## Results

The sweat and saliva samples were obtained from a total of 138 people at the Anticipa Health Center, Hermosillo, Sonora (Supplementary Material 2). Of the 138 samples of sweat, 69 were positive to SARS-CoV-2, and 69 were negative. In the case of saliva, 128 samples were obtained, from which 54 were positive to the virus and 74 were negative. The whole sample comprised 59% women and 41% men. The group of positive people comprised 59% women and 41% men, and the group of negative people was 42% women and 58% men. There were no differences between the COVID-19-positive and COVID-19-negative groups in the proportion of women (Fisher's exact test, difference between proportions = −0.02, p = 1) and men (Fisher's exact test, difference between proportions = −0.008, *p* = 1). The age range of the whole sample was between 18 and 60 years, with 37 ± 10 years for women and 38 ± 12 years for men. The age in the positive group was 39 ± 11 years for women and 37 ± 18 years for men; there was not a significant difference in age between the sexes (Student's *t*_0.05_ = −0.49, *p* = 0.62). The age in the negative group was 35 ± 9 years for women and 39 ± 11 years for men; there was not a significant difference in age between the sexes (Student's *t*_0.05_ = 1.27, p = 0.21). There were no differences in the mean age between the COVID-19-positive and COVID-19-negative groups (Student's *t*_0.05_ = −0.07, *p* = 0.94). The characteristics of the dogs exposed to sweat and saliva of COVID-19-positive and COVID-19-negative patients are presented in Table 1.

**Table 1:** Details of the six fully trained canine participants exposed to sweat and saliva samples of COVID-19-positive and COVID-19-negative humans.

**Name**	**Sex**	**Age (years)**	**Breed**	**Specialty**
Sam	Male	2	German Shepherd	Green dog^*^
Leia	Female	1	Golden retriever	Epilepsy
Mike	Male	2	German Shepherd	Green dog
Harry	Male	2	Belgian Malinois	Green dog
Krilling	Male	1	Belgian Malinois	Green dog
Spaidy	Male	2	Belgian Malinois	Green dog

* *A green dog is one that had not been trained previously to detect any kind of odor*.

Table 2 shows the sensitivity and specificity for the four dogs exposed to sweat samples compared with the results of RT-PCR and the antigen test. Sam and Leia had a marginal performance and their 95% CI overlapped the randomness region (50%). In contrast, Mike and Harry had sensitivity values of 76 and 80%, respectively, and their 95% CI were far from the randomness region. The specificity of Sam and Leia also overlapped the randomness region. In contrast, the specificity values for Mike and Harry were 75 and 88%, respectively, and their 95% CI did not overlap the randomness region.

**Table 2:** Sensitivity and specificity of the four dogs trained to detect COVID-19 from the sweat of positive and negative people compared with the reference tests (Antigen test and RT-PCR).

**Name**	***n***	**Sensitivity (%)**	**95% CI**	**Specificity (%)**	**95% CI**	**PPV** **(%)**	**95% CI**	**NPV** **(%)**	**95% CI**
Sam	132	58	45–71	69	57–80	61	48–74	67	55–77
Leia	132	60	47–72	64	52–76	62	49–74	62	50–74
Mike	124	76	63–86	75	63–85	74	61–84	78	66–87
Harry	95	80	66–91	88	75–95	86	72–95	83	70–92

*CI, confidence interval; n, number of trials; NPV, negative predictive value; PPV, positive predictive value; RT-PCR, reverse transcription polymerase chain reaction*.

Table 3 shows the sensitivity and specificity for the three dogs exposed to saliva samples compared with the results of RT-PCR and the antigen test. The dogs had sensitivity values between 70 and 78%, and for Spaidy and Krilling, their 95% CI did not overlap the randomness region. In contrast, the 95% CI of Leia overlapped the randomness region. The specificity for the three dogs followed a similar pattern, where the 95% CI for Spaidy and Krilling did not overlap the randomness region, while the one for Leia did.

**Table 3:** Sensitivity and specificity of the three dogs trained to detect COVID-19 from the saliva of positive and negative people compared with the reference tests (Antigen test and RT-PCR).

**Name**	***n***	**Sensitivity (%)**	**95% CI**	**Specificity (%)**	**95% CI**	**PPV** **(%)**	**95% CI**	**NPV** **(%)**	**95% CI**
Spaidy	138	70	56–82	69	58–78	58	45–70	79	68–87
Krilling	138	78	65–89	69	58–78	59	46–71	85	75–92
Leia	32	73	45–92	53	28–77	58	33–80	69	39–91

*CI, confidence interval; n, number of trials; NPV, negative predictive value; PPV, positive predictive value; RT-PCR, reverse transcription polymerase chain reaction*.

## Discussion

We originally hypothesized that our training procedure enables dogs to detect people with COVID-19 with a sensitivity and specificity significantly different from random. In that sense, all the dogs, with the exception of Sam and Leia for sweat (Table 2) and Leia for saliva (Table 3), had sensitivities significantly different from random. This was evident from the 95% CI for Mike and Harry (Table 2) and for Spaidy and Krilling (Table 3). Thus, we partially proved our hypothesis as correct. Moreover, the performance of the dogs was outstanding in view of the challenging circumstance in the field study: the pieces of gauze were exposed for no more than 5 min to axillary sweat or saliva. However, it is also evident from Table 2 that with the exception of Harry's 95% CI, none of the remaining dogs reached a sensitivity of ≥80%. It is desirable to reach or surpass this value to meet the World Health Organization (WHO) requirement for validation of antigen tests (22). If this sensitivity level is reached, then COVID-19-sniffing dogs could be considered at least as sensitive as the antigen tests currently available. In the case of specificity, the results for the dogs were not good: None of our dogs reached the ≥97% threshold established by the WHO for validation of antigen tests (22). Two mutually exclusive explanations for this result that together with other variables that could have affected the dogs' performance are presented below.

Our results suggested that the age range and sex of the people participating in the study were not relevant for the performance of the dogs. Although the age range was wide (18–60 years), the mean ages of women and men were very similar in COVID-19-positive and COVID-19-negative groups. There were no significant differences between the mean values of age of individuals or the proportion of females and males in the COVID-19-positive and COVID-19-negative groups. In addition, 97% of the people who provided samples came from just one place, the Anticipa Health Center in Hermosillo. Consequently, these similarities suggest that the samples of infected and non-infected people came from the same population and that both groups were comparable. This is important because, as mentioned by Grandjean et al. (14), significant differences in age between the groups being compared (infected vs. non-infected) cast doubts about the influence that this variable could have on the dog's performance to detect COVID-19-infected people, because body smell changes with age (23, 24). With respect to gender, we did not find significant differences between infected and non-infected individuals; hence, we met the comparability criterion for detection dog studies recommended by Edwards et al. (25).

Because most of our dogs did not overlap the randomness region (50%), we considered that the procedure for training them to detect people with COVID-19 was successful. The training procedure of the first generation of dogs lasted 4.5 months and was based on dog training procedures for the detection of narcotics, explosives, and epilepsy. The results showed that in 4.5 months, 50% of the dogs were able to reach sensitivity levels between 75 and 83%, and the other 50% reached sensitivity levels between 54 and 67% in the laboratory. Here, it is important to mention that with the exception of Leia, all other dogs were “green dogs” from narcotic line without previous experience in detection. Moreover, this is the first generation of dogs trained to detect COVID-19 in Mexico and, clearly, we dealt with a learning curve. This is especially noticeable when we compare our results with those of more experienced research groups who have reported a training time for detection of COVID-19 between 1.75 (26) and 3.75 months (27). This is certainly a matter of experience because a second generation of dogs has been trained in 2 months and are almost ready for their first field experience.

Based on the experience acquired by other groups working with dogs for COVID-19 detection and our own experience, we suggest that the training of dogs can be improved and the training periods shortened by: (1) using pseudo-scents (training aids in which the true material—SARS-CoV-2 in this case—is not part of the compound) (28), but only some of the chemical compounds produced by the human in response to the virus. These types of aids are commonly used during dog training for detection of bombs, narcotics, or human cadavers (29). In the specific case of COVID-19 sniffing dogs, there is a need of further scientific evidence on whether these aids increase the detection capacity of the dogs. However, pseudo-scents are considered as very useful for training dogs for the detection of narcotics such as cocaine (30). (2) The use of trained dogs which apparently could increase the speed at which they become proficient in the detection of COVID-19 to a few weeks compared with several months to over a year for juvenile green dogs without detection training. The previous point seems to be critical, but whether dogs belong to pure or mixed dog breeds seems to be irrelevant (14, 27, 31), and (3) exposing the dogs to samples of different respiratory tract infections together with SARS-CoV-2 samples during their training period as ten Haggen et al. (13) have recently demonstrated.

The present study is one of the first field experiences exposing trained dogs to sweat and saliva samples immediately after they have been acquired from patients. Unfortunately, the short time we were allowed by the health center to expose the pieces of gauze to the armpit and mouths of the patients probably negatively affected the performance of the dogs (Tables 2, 3). Thus, our results suggest the need to keep the pieces of gauze in the patients' armpits or mouths for at least 10 min, as recommended by Grandjean et al. (14), and ideally 20 min as other authors have done (32). Even under these challenging circumstances, the dogs performed relatively well. In fact, one of our sweat-sniffing dogs (Harry) reached 80% sensitivity, with a 95% CI that was far from the randomness region. This is considered a good result because it falls within the range requested by the WHO for the performance of the antigen test (22). Mike, another sweat-sniffing dog, was very near this sensitivity value (76% sensitivity; Table 2) with his 95% CI overlapping the 80% sensitivity value. The other two sweat-sniffing dogs did not reach 80% sensitivity with their 95% CI. Clearly, these were the dogs most affected by the short exposure time of the pieces of gauze to the patients' sweat. In the case of saliva (Table 3), neither Spaidy nor Krilling reached 80% sensitivity. However, the 95% CI of both saliva dogs overlapped this sensitivity value. Thus, it is highly probable that if the exposure time increased, the dogs would have a better performance. In the case of Leia, her sensitivity to saliva samples reached 73%. However, her 95% CI was highly variable (0.45–0.92), overlapping the randomness region. This was probably due to the low number of trials (32) to which she was exposed. Clearly, with more trials, the 95% CI values become more reliable as in the case of all other dogs in Tables 2, 3. Thus, the experience acquired in this case suggest that the number of trials should be at least 100, but it would be much better well beyond 100 trials to reach a reliable estimation. Note that this number is similar to the one obtained by the study power analysis (*n* = 94).

With respect to specificity, the results in Tables 2, 3 suggest that the dogs were affected negatively by the short time we were allowed by the health center to expose the pieces of gauze to the patients' armpits and mouths. The most likely explanation for this low level of specificity is that the dogs are not identifying COVID-19 only, but also other respiratory infections. All the people who participated in the present study were there because they were experiencing symptoms of an infection of the respiratory tract and looking for a SARS-CoV-2 test. It is likely that many of these people were infected with respiratory infections other than COVID-19 as shown by the negative RT-PCR and antigen test results. It may be prudent to consider that during their training, the dogs should be exposed to other respiratory infections at the same time they are exposed to SARS-CoV-2 as ten Haggen et al. (13) have recently suggested. Thus, it is highly possible that the dogs were identifying chemical clues related to a general response of the patients to those infections (including COVID-19), as D'Aniello et al. (33) proposed. These authors pointed out that all previous experiences published have been experimental exposures of the dogs to sweat, saliva, or tracheobronchial secretions of healthy or sick people with COVID-19. Thus, they emphasized the need to expose the dogs to real-life conditions to determine their performance, and they predicted that a decrease in the performance of the dogs could be expected due to the many odors present in hospitals or health centers. We attributed the low level of specificity reached by our dogs to the confounding variables generated by different infections of the respiratory tract in the people present in the Anticipa Health Center. Grandjean et al. (32) have also suggested that several health conditions of the positive patients could act as confounding factors for the dogs' performance to detect COVID-19. We also concur with Essler et al. (15) and D'Aniello et al. (33) on the need for a careful study of VOC produced by the people with COVID-19 and non-infected people. However, a further concern that should be raised is the need for specific identification of the respiratory infections of the false-positive people marked by the dogs. This information would allow matching the identity of the infection with the profile of VOC produced by the patients to better tune the dogs' training.

In addition to the low specificity of the dogs due to the possible detection of other respiratory infections, there are other confounding factors that should be addressed in future work. Apparently, working with the dogs every day 5 days a week with two sessions per day negatively affects the dog's performance. Thus, an additional potential explanation for the poor performance of our dogs could be related to this fact. In contrast, Mendel et al. (34) obtained high positive predictive values (73.7–93.9) exposing the dogs three times per week to COVID-19 samples. This point deserves careful consideration because it certainly increases the number of dogs needed for detection. Another problem to solve is the immediate access to the COVID-19 test result of the person being sniffed to know whether the dog should be rewarded. In fact, in our study the RT-PCR results were delayed from 2 days up to 2 weeks. We solved this problem partially by asking the people for a sample for an antigen test. However, there are two additional problems with this procedure. First, antigen tests are not as sensitive as the RT-PCR test, especially if the person has an early infection and is still building up the viral load. Second, the people are reluctant to provide another nasopharyngeal sample because it is painful or at least uncomfortable, especially after the sample extraction for the RT-PCR. An alternative is to use DNA/RNA shield saliva/sputum collection kits to collect samples of symptomatic and asymptomatic people, combined with rapid molecular tests such as RT-LAMP. These seem to be easy ways to obtain reliable samples to detect the virus (3, 34).

An advantage of the use of dogs is their capacity to detect infected people before RT-PCR or the antigen test. If a patient had loss of taste and/or smell and negative RT-PCR and antigen test, but the dogs alerted the sample was positive, we decided to follow up with the patient by telephone, allowing us to know their health status and, if a second test was carried out, to know its result. Due to the follow-up of the patients, six cases were detected that originally had a negative RT-PCR and the dogs marked the patient as positive. Two to five days later, the patients who were followed up underwent a second lab test. Two of those cases were checked with the antigen test, three had RT-PCR, and one case had an antibody test. The results of these six follow-up cases were positive for SARS-CoV-2, agreeing with the results of the dogs. Similar results have been reported by Grandjean et al. (14) and Carvalho et al. (35), who found two people and one person, respectively, who the dogs marked as positive, but their RT-PCR tests were negative, and a few days later they had the test again and were positive. Thus, our results and those of the researchers mentioned above suggest dogs' performances are likely better than reported because of the somewhat flawed RT-PCR reference standard test used for comparison. Further, the antigen tests are much less accurate than the RT-PCR test, so dog “errors” based on these tests are even more unreliable and can lead to poorer dog sniffing results [see (36) for an excellent comparison of the performance of RT-PCR and antigen tests for detection of SARS-CoV-2]. Certainly, this is an area that deserves careful attention.

One of the main ethical concerns regarding the use of sniffing dogs is the potential risk for them to become infected due to exposure to COVID-19-positive samples. To try to diminish this risk, we used stainless-steel salt shakers that act as physical barrier to avoid compromising the dogs' health. Fathizadeh et al. (37) showed that sweat of the hands of people with COVID-19 did not contain the virus. However, there is no information published on the presence of the virus in sweat of other human body parts. Therefore, the use of salt shakers appears to be a reasonable option for the dogs to sniff only VOC without physical contact with the gauze impregnated with the sweat or saliva of infected patients. Other methods of inactivation of the samples of SARS-CoV-2 such as beta-propiolactone (3), NP-40 detergent and heat (15) have been used. In addition, it has been demonstrated that the use of UV radiation to inactivate SARS-CoV-2 does not change the VOC composition of face masks of infected people exposed to dogs (34). Thus, apparently, these preventive sample treatments do not affect the quality of the samples at all. The use of these methods to prevent a dog's infection is crucial since they can become infected with the virus, even when the published evidence suggests that their infection level can be from low (38, 39) to no infection at all (40).

In conclusion, due to the challenging conditions of only 3 min exposure of the pieces of gauze to axillary sweat and saliva, only one of our dogs reached 80% sensitivity. However, another sweat-sniffing dog (Mike) and two saliva-sniffing dogs (Spaidy and Krilling) had 95% CI that did not overlap the random region (50% of sensitivity). These results seem promising even though only one dog reached the sensitivity range requested by the WHO for the performance of an antigen test (22). In the case of specificity, the results were not favorable, but apparently the dogs are able to detect people sick not only with COVID-19, but also other kind of respiratory diseases. We are almost certain that if the pieces of gauze were left under the armpit or the buccal swabs were left in the mouth for at least 10 min, the dogs would show improved sensitivity and specificity. This eventuality is very important because if the dogs reach the sensitivity and specificity ranges required by the WHO for the performance of antigen tests, they could become a crucial ally alongside other molecular and antigen test to control the COVID-19 pandemic. This claim is based on the fact that if we are able to detect COVID-19 in a fast and reliable way, we could isolate the infected individuals and thus decrease transmission dramatically. Very strong support for this view and for the role of COVID-19-sniffing dogs is the mathematical modeling simulations of Larremore et al. (41). These authors suggested that effective screening of COVID-19 depends largely on the frequency of testing and the speed of reporting rather than high test sensitivity. If this is so, then COVID-19-sniffing dogs have a bright future, because even if their sensitivity and specificity levels decrease in the field, they still can provide very fast and reliable results each day for several years.

## Data Availability Statement

Datasets are available on request. The raw data supporting the conclusions of this article will be made available by JM-T (jmancilla@obi-k19.com) without undue reservation.

## Ethics Statement

The studies involving human participants and animal study were reviewed and approved by Comité de Ética en Investigación de la Universidad de Sonora. The patients/participants provided their written informed consent to participate in this study.

## Author Contributions

JM-T and VV-M conceptualized the study, drafted the manuscript, tables, and prepared the final version. VL-E, AO, RO-C, RR-Z, BM-C, JB-C, IR-L, CG-B, and AN-G collected and compiled data. All authors contributed to data interpretation, critical revision, and approved the final manuscript for submission.

## Funding

This work was supported by Mr. Juan Manuel Mancilla-Leal and Purina Pet Care. Purina Pet Care was not involved in the study design, collection, analysis, interpretation of data, the writing of this article or the decision to submit it for publication.

## Conflict of Interest

JM-T, VL-E, and AO were employed by Canine Training Center Obi-K19. The remaining authors declare that the research was conducted in the absence of any commercial or financial relationships that could be construed as a potential conflict of interest.

## Publisher's Note

All claims expressed in this article are solely those of the authors and do not necessarily represent those of their affiliated organizations, or those of the publisher, the editors and the reviewers. Any product that may be evaluated in this article, or claim that may be made by its manufacturer, is not guaranteed or endorsed by the publisher.
