# Supplementary material for: Dogs Detecting COVID-19 From Sweat and Saliva of Positive People: A Field Experience in Mexico
Source: Front Med (Lausanne). 2022 Apr 1;9:837053. doi: 10.3389/fmed.2022.837053 (PMC9012113; doi:10.3389/fmed.2022.837053)
Supplement: Supplementary file 1 [file Data_Sheet_1.PDF]

Centro Canino de Detección de Enfermedad

OBI K-19

INFORMED CONSENT LETTER

In accordance with the provisions contained in the General Health Law, Fifth Title "Health Research", Sole Chapter, article 100, section IV; as well as the Regulation of the General Health Law on Research for Health, Second Title "Of the Ethical Aspects of Research in Human Beings" Chapter I, Common Provisions, Article 13 which states that in all research in which the human being is the subject of study, the criteria of respect for their dignity and the protection of their rights and well-being must prevail, articles 14 section V, 20, 21 and 22 of this Regulation; and, in accordance with the ethical principles contained in the Declaration of Helsinki, it has been explained to me and informed that:

I. It has been explained to me that I possibly suffer from the SARS-CoV-2 (COVID-19) viral infection and that I am proposed to participate in the research intiative to test the detection of this virus using body fluids as a possible alternative to detection by RT -PCR.

II. I have been informed that 5 sweat samples, 5 saliva samples and 1 urine sample of 60 ml will be taken, this is in addition to the study that requires the RT-PCR test. In addition, personal data related to my age, diet, use of medications, etc. will be obtained from me.

III. The results of this study will help to better determine the presence of SARS-CoV-2 (COVID-19) in muy case and in other patients.

IV. I have been assured that I can ask questions about the study and my participation to the best of my satisfaction

V. I authorize the publication of the results of my study on the condition that professional secrecy will be mantained at all times and that my name will not be published or my identity will be revealed.

VI. The researchers are committed to providing me with up-to-date information obtained during the study, although this may contradict the results obtained in the RT-PCR test.

VII. Laboratory sudies (RT-PCR) and detection using dogs will be covered by the Anticipa Center and the Research Project "OBI: canines against COVID

With date ______________________________, having understood the above and once all the doubts regarding my participation in the project have been clarified I : _________________________________ _______________________________ with patient number _________________ agree to participate in the study entitled : "OBI: Caninos contra COVID".

Patient´s name and signature ____

Name and signature of witness ____

Contact:
Juan Manuel Mancilla Tapia
jmancilla@hourslt.com

OBI·K19
Centro de Biodetección K-9

HORUS LT

MEDICAL AND CONSUMPTION HISTORY

PLEASE FILL AND MARK AS NECESSARY

Medical condition	Yes	No
Cancer		
Hypertension		
Diabetes		
Respiratory conditions (Asthma , tuberculosis, etc.)		
Epilepsy		
Alcohol last 24 hr		
Recreational drugs or CBD last 24 hr		

Other:____

CONSUMPTION

Consumption	Daily or more than 3 times per week	Occasionally	None	Last 24 hr
Alcohol				
Smoking				
Recreative drugs or CBD				

SYMPTOMS:

Diarrhea	Fever	Dry cough	Nausea	Loss of taste	Loss of smell	Headache	Throat pain	Chest pain	Body pain	Fatigue	Runny nose

Symptoms and prescriptions

Days with symptoms	Days
**Days with prescriptions**	**Days**

Menstrual period Yes No

Gender: Female/Male
Age:

Thank you very much for your help
